# Analysis of ancient human mitochondrial DNA from the Xiaohe cemetery: insights into prehistoric population movements in the Tarim Basin, China

**DOI:** 10.1186/s12863-015-0237-5

**Published:** 2015-07-08

**Authors:** Chunxiang Li, Chao Ning, Erika Hagelberg, Hongjie Li, Yongbin Zhao, Wenying Li, Idelisi Abuduresule, Hong Zhu, Hui Zhou

**Affiliations:** College of Life Science, Jilin University, Changchun, 130023 P. R. China; Ancient DNA Laboratory, Research Center for Chinese Frontier Archaeology, Jilin University, Changchun, 130012 P. R. China; Department of Biosciences, University of Oslo, 0316 Oslo, Norway; Life Science College, Jilin Normal University, Siping, 136000 P. R.China; Xinjiang Cultural Relics and Archaeology Institute, Ürümchi, 830000 P. R. China

**Keywords:** Ancient DNA, Mummies, Human populations, Tarim Basin, Mitochondrial DNA

## Abstract

**Background:**

The Tarim Basin in western China, known for its amazingly well-preserved mummies, has been for thousands of years an important crossroad between the eastern and western parts of Eurasia. Despite its key position in communications and migration, and highly diverse peoples, languages and cultures, its prehistory is poorly understood. To shed light on the origin of the populations of the Tarim Basin, we analysed mitochondrial DNA polymorphisms in human skeletal remains excavated from the Xiaohe cemetery, used by the local community between 4000 and 3500 years before present, and possibly representing some of the earliest settlers.

**Results:**

Xiaohe people carried a wide variety of maternal lineages, including West Eurasian lineages H, K, U5, U7, U2e, T, R*, East Eurasian lineages B, C4, C5, D, G2a and Indian lineage M5.

**Conclusion:**

Our results indicate that the people of the Tarim Basin had a diverse maternal ancestry, with origins in Europe, central/eastern Siberia and southern/western Asia. These findings, together with information on the cultural context of the Xiaohe cemetery, can be used to test contrasting hypotheses of route of settlement into the Tarim Basin.

**Electronic supplementary material:**

The online version of this article (doi:10.1186/s12863-015-0237-5) contains supplementary material, which is available to authorized users.

## Background

**Fig. 1:**
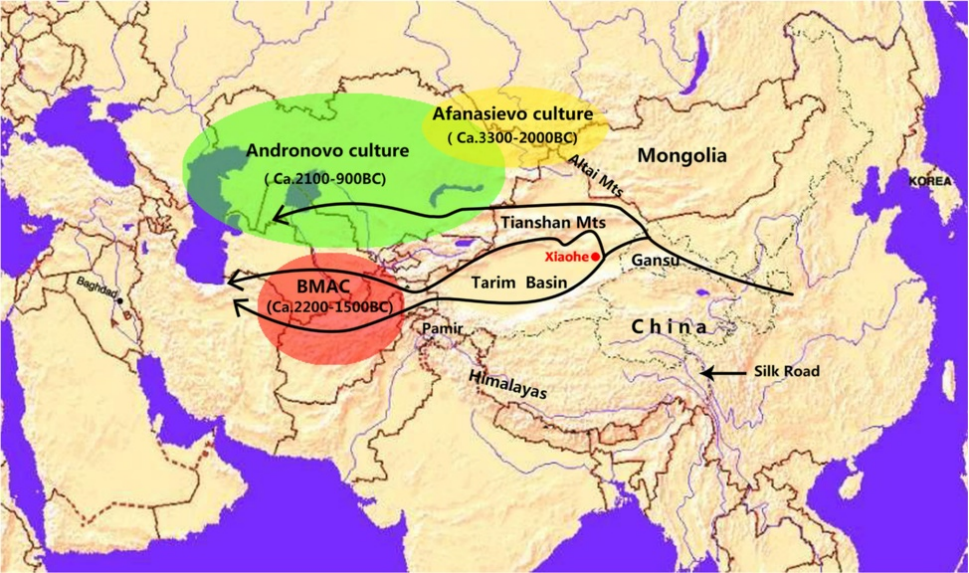
Map of Eurasia showing the location of the Xiaohe cemetery, the Tarim Basin, the ancient Silk Road routes and the areas occupied by cultures associated with the settlement of the Tarim Basin. This figure is drawn according to literatures

The Tarim Basin in the Xinjiang region of China is situated on the Silk Road, the collection of ancient trade routes that for several millennia linked China to the Mediterranean (Fig. 1). The present-day inhabitants of the Tarim Basin are highly diverse both culturally and biologically as a result of extensive movements of peoples and cultural exchanges between east and west Eurasia [1–3]. Archaeological and anthropological investigations have helped to formulate two main theories to account for the origin of the populations in the Tarim Basin [4–12]. The first, so-called “steppe hypothesis”, maintains that the Tarim region experienced at least two population influxes from the Russo-Kazakh steppe. The earliest settlers may have been nomadic herders of the Afanasievo culture (ca. 3300–2000 B.C.), a primarily pastoralist culture derived from the Yamna culture of the Pontic-Caspian region and distributed in the Eastern Kazakhstan, Altai, and Minusinsk regions of the steppe north of the Tarim Basin (Fig. 1) [9, 12–15]. This view is based on the numerous similarities between the material culture, burial rituals and skeletal traits of the Afanasievo culture and the earliest Bronze Age sites in the Tarim Basin, such as Gumugou (ca. 3800 BP), one of the oldest sites with human burials in Xinjiang [8, 9, 11, 12, 16]. These first settlers were followed by people of the Late Bronze Age Andronovo cultural complex (ca. 2100–900 B.C.), another pastoralist culture derived from the Yamna culture, primarily distributed in the Pamirs, the Ferghana Valley, Kazakhstan, and the Minusinsk/Altai region (Fig. 1) [8, 9, 11, 12, 15, 16]. This is signaled by the introduction of new material culture, clothing styles and burial customs around 1200 B.C. The second model, known as the “Bactrian oasis hypothesis”, also postulates a two-step settlement of the Tarim Basin in the Bronze Age, but maintains that the first settlers were farmers of the Bactria–Margiana Archaeological Complex (or BMAC, also known as the Oxus civilization) (ca. 2200–1500 B.C.) west of Xinjiang in Uzbekistan (north Bactria), Afghanistan (south Bactria), and Turkmenistan [17], followed later by the Andronovo people from the northwest (Fig. 1) [5, 7]. This model emphasises the environmental similarities between the Xinjiang and Central Asian desert basins, and suggests that certain features, including the irrigation systems, wheat remains, woolen textiles, bones of sheep and goats, and traces of the medicinal plant Ephedra found in Xinjiang could be evidence of links with the Oxus civilization [5, 7, 16]. These contrasting models can be tested using DNA recovered from archaeological bones. Previous genetic evidence on the origin of the earliest settlers was based on the analysis of mtDNA from burials at the Gumugou cemetery in the eastern edge of the Tarim Basin. In that study, researchers sequenced the first mtDNA hypervariable region (HVRI), but the results were inconclusive [18]. The discovery of another Bronze Age site of a similar age to Gumugou, with many well-preserved mummies, including individuals with European facial features, provided a unique opportunity to obtain genetic evidence about the first settlers of the Tarim Basin [19–21].

**Fig. 2:**
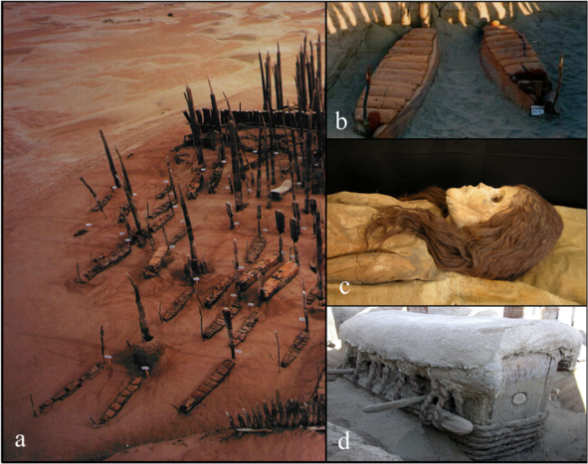
**a** Fourth layer of the Xiaohe cemetery showing a large number of large phallus and vulva posts; **b** A well-preserved boat coffin; **c** Female mummy with European features; **d** Double-layered coffin excavated from the Xiaohe cemetery

We describe here the analysis of mtDNA from human remains recovered from the Xiaohe tomb complex, an important Bronze Age site in the eastern edge of the Tarim Basin (40°20′11″N, 88°40′20.3″E) (Fig. 1). Discovered originally in 1934 by the Swedish archaeologist Folke Bergman, it was subsequently lost, but rediscovered in 2000 by a team from the Xinjiang Archaeological Institute using global positioning equipment. The cemetery was excavated between 2002 and 2005, and consisted of five strata with radiocarbon dates ranging from 4000 to 3500 years before present (^14^C yBP) [19, 22]. The site has many notable features, including numerous large phallus and vulva posts made of poplar, striking wooden human figures and masks, well-preserved boat coffins, leather hides, wheat and millet grains, and many artifacts (Fig. 2). Importantly, it contains the oldest and best-preserved mummies so far discovered in the Tarim Basin, possible those of the earliest people to settle the region. Genetic analysis of these mummies can provide data to elucidate the affinities of the earliest inhabitants, and help understand later patterns of human migration in the Eurasian continent.

The necropolis consisted of five layers of burials spanning half a millennium, offering the opportunity to determine the extent of interactions between the people of Xiaohe and other populations after the original settlement of the Tarim Basin. Did the people remain comparatively isolated or did they intermarry with newcomers? In an earlier study, we analysed DNA recovered from the deepest and oldest layer of burials of the Xiaohe site, the fifth layer, corresponding to the earliest inhabitants. Our results revealed that the first settlers carried both European and central Siberian maternal lineages. These findings agreed with the archaeological evidence for a close connection to the Afanasievo culture of the steppe north of the Tarim Basin, in other words with the “steppe hypothesis” [23]. We describe here the analysis of the maternal lineages of individuals recovered from the remaining four burial layers, and discuss the results in the context of the contrasting views on the settlement and migration patterns of the Tarim Basin.

## Methods

### Bone samples

The human remains excavated from the Xiaohe burial complex exhibited excellent preservation by virtue of the dry, sandy, and well drained soil, which is both alkaline and high in salt. The cemetery, consisting of 167 graves, was excavated by the Xinjiang Provincial Institute of Cultural Relics and Archaeology, with permission from the State Administration of Cultural Heritage, who has control of archaeological excavations in China. After recording and photographing, the skeletal remains of 92 well-preserved individuals were placed in cardboard boxes, together with the surrounding sandy soil, and sent to the ancient DNA laboratory of Jilin University, where they were stored in a cool and dry environment. Bone and tooth samples were collected by two skilled staff members, wearing disposable gloves and face masks. Thirty individuals, representing the oldest layer, were analysed in a previous study [23]. The present study included 28 individuals of the fourth layer, seven from the third layer, and 27 from layers 1–2, among which 22 human samples were scattered on the surface of sand due to the burials of the uppermost two layers were damaged by looters and weathering. Teeth and bone were taken from each individual whenever possible. Details of the samples are included in the electronic supplement (Additional file 1: Table S1).

Bones were processed and DNA extracted as described previously [23], with the inclusion of an extraction blank for every three ancient samples.

### DNA authentication and prevention of contamination

Strict precautions were taken to avoid contamination by modern DNA. Ancient DNA degradation and potential contamination were monitored as described by Gilbert *et al.* [24]. In brief, DNA extractions, and steps performed before polymerase chain reaction amplification (PCR), were performed in a building remote from the post-PCR laboratory, in a laboratory dedicated exclusively to ancient DNA research. The laboratory was equipped with positive air pressure, and rooms were irradiated overnight with UV light (254 nm). Surfaces were cleaned frequently with DNA Off. Extraction and amplification blanks were included in every PCR assay in order to detect any potential contamination from sample processing or reagents. Multiple extractions and amplifications from the same individual were undertaken at different times and from two different parts of the skeleton, such as bone and tooth, to detect artefactual sequences due to cross-contamination, pre-lab contamination, DNA damage or jumping PCR events. Partly samples were chosen randomly to do independent repetition in our new lab by one different laboratory member in order to detect the contamination in laboratory environment. PCR amplicons of six of the ancient DNA extracts were cloned to check for potential heterogeneity in the amplification products due to contamination, DNA damage, or jumping PCR. MtDNA amplicons of different sizes were analysed to investigate the inverse correlation between amplicon size and amplification efficiency. Ancient DNA from cattle remains, found at the same site, was isolated using the same procedure as for the human ancient DNA, providing an additional control for contamination. Lastly, the DNA types of the archaeologists and laboratory personnel were compared to the experimental results to check for potential contamination, as described in a previous study [23].

### DNA quantification and PCR amplification

Three ancient extracts were chosen at random to quantify amplifiable mtDNA of four different fragment sizes, namely 138, 209, 235 and 393 base pairs (bp), using a GenAmp 5700 Sequence Detector (Applied Biosystems, USA). qPCR amplification was performed in 25 μL reactions containing 1X SYBR Green PCR Master Mix (Applied Biosystems, USA), 0.5 μM each primer, 2 mM BSA (Takara, Japan) and 5 μL DNA extract. The specificity of primers was validated using modern DNA, and a single peak was observed when monitoring post-PCR melt curve for all fragments, indicating specific binding. The Mitochondrial sequence polymorphisms (HVRI) were analysed by amplifying a segment spanning nucleotide positions 16035–16409, using two overlapping primer pairs. In addition, several mtDNA coding region polymorphisms diagnostic for major branches of the human mtDNA tree were typed, as follows: Haplogroups (Hgs) R (12705C), UK (12308G), HV (14766C), H (7028C), R1 (4917G), R11 (10031C), M5 (1888A), M25 (15928A), C4 (11969A) and G (4833G) were identified by direct sequencing. Hgs M (10400 T), C (14318C), T(15607G) and D (5178A) were analysed by the PCR product-length polymorphism method . Haplogroup (Hg) B was identified on the basis of the 9-bp deletion at position 8280 [25–27]. A table of the primers is included in the electronic supplement to this paper (Additional file 2: Table S2). The sex of the Xiaohe individuals was determined by PCR of the sexually dimorphic amelogenin gene [28, 29]. PCR amplifications were performed in 20 μL reactions, as described previously [23].

### DNA cloning and sequencing

To investigate potential contamination of the PCR amplicons, DNA amplified from six individuals chosen at random was cloned using the pGEM-T Easy Vector System I (Promega, USA). Eight white clones of each PCR fragment were sequenced using M13 primers. Cycle sequencing was performed as described previously [23], and the sequences analysed using an ABI310 Genetic Analyzer (Applied Biosystems, USA), following the instructions of the manufacturer.

### Data analysis

Sequence alignments were performed using ClustalX 1.8 software, followed by manual editing. Published literature and the Genbank database were searched to identify shared sequences. The sequences were subject to statistical analysis, including 20 additional sequences previously obtained from the fifth and lowest layer of the Xiaohe cemetery. Haplotype diversity was investigated using DnaSPv5 (http://www.softpedia.com/get/Science-CAD/DnaSP.shtml). The results for layers 1–3 were pooled, as the sample was small and the layers had been commingled by grave looters. The Networks of four mtDNA haplogroups were constructed by Network software ver. 4.6.1.3 (www.fluxus-engineering.com) using the median-joining method. The multidimensional scaling (MDS) was conducted using Arlequin 3.5 software (http://cmpg.unibe.ch/software/arlequin3/) and SPSS16.0 (USA). Principal Component Analysis (PCA) was performed with SPSS 16.0 software (USA), using a haplogroup frequency database of ancient and present-day populations, with 17 different haplogroups (Additional file 3: Table S3). Fifteen of these were Hgs A, B, C, D, Z, F, G, N9, HV, U, K, W, X, R and TJ, while a further seven east Eurasian Hgs (E, M7, M8, M9, M10, M11 and M13) were pooled into one group, and an additional four west Eurasian Hgs (I, N1a, N1b and N*), were pooled into a final group.

## Results

### Authentication of results

A total of 42 reproducible mtDNA sequences (345 bp) were obtained from 62 individual sets of human remains, after discarding 20 samples due to failed amplification or lack of reproducibility. Six of the 42 sequences matched with two archaeologists and one laboratory member were also removed from the study, even though they yielded consistent results through multiple independent extractions. The remaining 36 sequences were inferred to be unambiguous and believable. The following criteria supported the authenticity of the results: (i) an inverse correlation between the size of the PCR amplicons and amplification efficiency (Additional file 4: Table S4); (ii) consistent consensus cloned sequences, although a small number of sites differed from the directly sequenced PCR products, possibly due to random *Taq* mis-incorporation or DNA damage. Miscoding lesions in clones of PCR products showed that cytosine → thymine changes characteristic of damaged ancient DNA were the most frequent changes in the Xiaohe individuals (Additional file 5: Figure S1); (iii) sex determination by molecular and morphological methods gave consistent results (Table 1); (iv) the mtDNA HVRI sequences corresponded to the key coding region SNPs defined by the mtDNA phylogenetic tree [26]; (v) analysis of cattle bones from the Xiaohe site using the human-specific primers did not reveal human DNA, implying the bones were free of human DNA and the extractions were done cleanly; (vi) the mtDNA sequences from multiple independent DNA extractions and using different samples (tooth, femur) were consistent (Additional file 6: Table S5). The 36 sequences accepted as genuine bone sequences have been submitted to GenBank, with accession numbers KF436896-KF436931.

**Table 1:** Result for mitochondrial DNA typing

Sample number	HVR-I sequence (np16050-16409), minus np 16000	mtDNA-Hg (HVR-I)	mtDNA-Hg (SNP)	Sex identification		
				Morphological	Molecular	
Upper layer(layers1-3)						
T18-1	51-223-362	D	D	male	male	C
T18-7	223-278-293-297-362	G2a	G	-	male	
T22-6	223-234-316-362	D	D	-	-	
T23-4	298-327	C4	C4	-	-	C
T24-7	298-327	C4	C4	male	male	
T24-12	129-223	M5	M5	male	male	
T28-5	192-256-270	U5a	U	-	male	C
T28-8	182C-183C-189-217-243-355	B5	B	-	male	C
T28-9	298-327	C4	C4	-	-	
T29-12	129-223-304	M	M	male	male	
T35-1	184-223-298-319	M	M	-	male	
MW	318T	U7	U	female	female	
M12	CRS	H	H	female	female	
M39	129-223-298-327	C4	C4	male	male	
M55	93-129-223-298-311-327	C4	C4	female	female	
M62	183C-189-224-256-311	K	K	male	male	
Fourth layer						
Bm1	223-298-309-327	C4	C4	female	female	Q
Bm2	223-298-309-327	C4	C4	female	female	
Bm5	126-292-294	T	T	female	female	
Bm9	51-129C-182C-183C-189-261-362	U2e	U	male	male	Q
Bm10	223-298-309-327	C4	C4	male	male	
BM18	223-288-298-327	C5	C	female	female	
Bm20	192-223-266-362	D	D	female	female	
Bm22	318 T	U7	U	male	male	
BM24	223-298-309-327	C4	C4	male	male	
Bm25	183C-189-261-311-390	R	R	male	male	
M70	223-298-309-327	C4	C4	male	male	
M73	172-183C-209-223-362	D	D	male	-	
M75	223-298-309-327	C4	C4	female	female	
M87	223-298-309-327	C4	C4	male	male	C
M89	298-327	C4	C4	female	-	
M93	298-327	C4	C4	female	-	
M95	192-256-270-291	U5a	U	male	-	
M99	223-298-309-327	C4	C4	female	female	
M129	223-298-327	C4	C4	male	male	
M130	223-298-309-327	C4	C4	male	male	C;Q

C: sample was cloned and sequenced; Q: sample was quantified; - (hyphen): sample did not amplify

### Mitochondrial DNA profiles and haplogroups

The 36 successfully typed individuals yielded 21 distinct mtDNA haplotypes, of which 18 could be assigned to 12 previously defined haplogroups [30–32] by means of HVRI and coding region polymorphisms (Table 1). The haplogroups were the west Eurasian H, K, T, U7, U5a, U2e, the east Eurasian B, C4, C5, D, G2a, and the Indian M5.

**Fig. 3:**
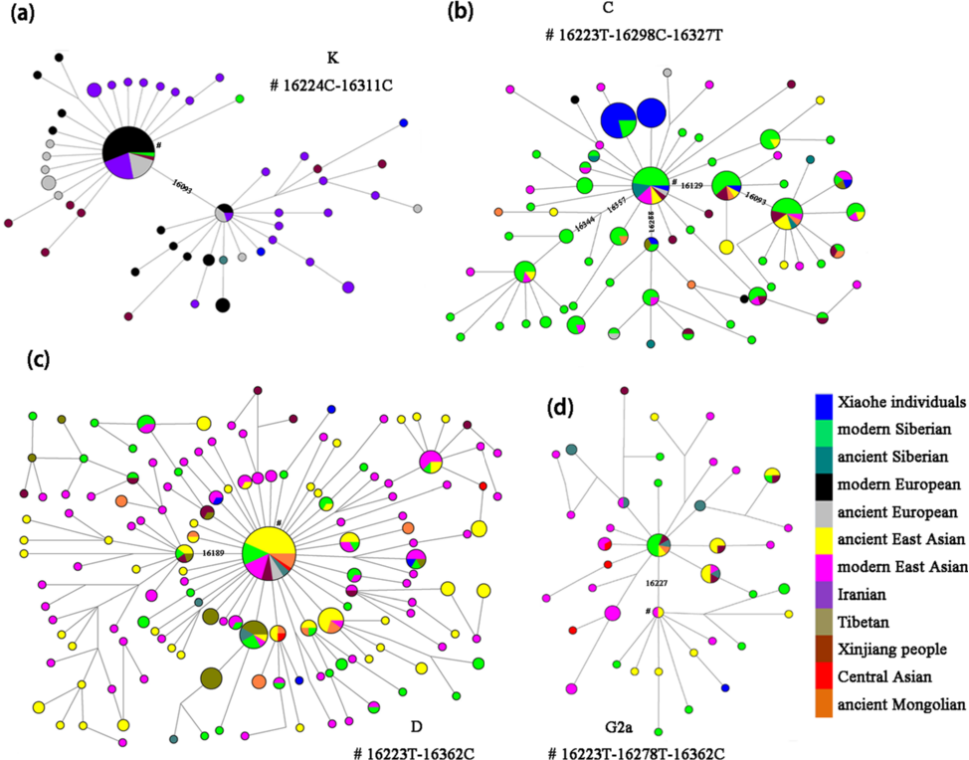
Median joining networks for mtDNA haplogroups K, C, D and G2a, based on HVS-I sequences between region np16050-16391. Circle areas are proportional to haplotype frequency. The length of the lines between nodes is proportional to the mutation steps. The diagnostic mutations used to classify the major branches are labeled on the line. The Number sign(#) and the following panels indicate the assumed root of each haplogroup

The west Eurasian haplogroups of the Xiaohe people were more diverse (Hd = 0.9722 versus Hd = 0.8585), but less abundant (9 individuals versus 26 individuals) than the East Eurasian haplogroups. The predominant lineage was UK, of which four different subhaplogroups were observed: one K, two U7, two U5a, and one U2e. One individual with Hg T and one individual with Hg H were detected. The latter carried the HVRI Cambridge Reference Sequence (CRS), very common in living Europeans [31, 33, 34]. This sequence has also been observed in ancient human remains of Neolithic Europe [35, 36], the Bronze Age in central Asia [37], as well as the Mongolian Altai Mountains [38], and the Iron Age in southern Siberia [39]. The T haplotype observed in Xiaohe is found exclusively in Europeans, with the exception of Iran in modern people, and found mostly as T2. It has also been observed in human remains of Neolithic Europe [36], the Eneolithic/Bronze Age in the Pontic Caspian steppe [40], and the Bronze Age in Kazakhstan [37]. No exact match was found for the Xiaohe K haplotype in our database. The network shows that it clusters into one subclade with the 16093 mutation, which is mainly distributed in Europe and Iran (Fig. 3a). Therefore, the K haplotype sequenced in Xiaohe is currently uninformative about population affinity. There are two U5a haplotypes observed in Xiaohe, the basal U5a*(16192 T-16256 T-16270 T) was found broadly in Europe and central Asia, while the derived U5a haplotype(16192 T-16256 T-16270 T-291 T) was found exclusively in Europe for modern people. These two sequences have also been found in Neolithic Europe [35, 41, 42]. U5a is a very ancient and important European haplogroup and is thought to have expanded eastward into central Siberia. It has been observed in human remains of the Neolithic in the Baikal regions and the Bronze Age in the Altai and Xinjiang [39, 43, 44]. The U2e sequence observed in Xiaohe did not match any sequence in our database, the most matching sequences (showing one to two np differences) were mainly found in Europe. U2e also was an ancient European lineage like U5, and had spread into Central Eurasia in the Bronze Age [31, 39, 44]. The presence of individuals of Hgs H, T, U5a and U2e in Xiaohe indicates maternal lineages with an ultimate origin in Europe. HgU7 is absent in many parts of Europe, but its frequency increases to >4 % in the Near East and up to 5 % in Pakistan, reaching almost 10 % in Iranians, and its highest frequency in Gujarat. U7 haplogroup probably originated in the region between Iran and Indian Gujarat [45–47]. The U7 variant observed in Xiaohe is currently found mostly in Iran, Europe and the Tibetan plateau. In addition, we found one individual with the Indian lineage M5 [48]. Nowadays, the M5 variant observed in this study is found mainly in south and southwest Asia. The presence of hgs U7 and M5 in the Xiaohe people suggests that populations of west/south Asia contributed to the gene pool of the Tarim Basin during the Bronze Age.

The most dominant east Eurasian haplogroup in the Xiaohe people was C, found in 18 of the 36 individuals (47 %) and associated with five distinct mtDNA C4 haplotypes and one C5 haplotype. Nine Xiaohe individuals carried the variant 16223-16298-16309-16327 and five carried the variant 16298–16327. The first of these variants, 16223-16298-16309-16327, has to our knowledge not been previously observed in ancient or living populations, while the variant 16298–16327 was only observed in present-day Siberia, although at low frequencies [49–51]. A variant characterised by substitutions 16223-16298-16327, observed in one Xiaohe individual, is found widely in present-day Eurasia, with the highest frequency in central/eastern Siberia. It also been detected in a number of ancient individuals, three from Neolithic central Siberia [43], one from northeast Siberia (3600 yBP) [52], six from northeast Europe (3500yBP) [37], twelve from the Bronze Age West Siberian Plain [53], one from southern Xinjing(2800-2011yBP) [54] and four from late Neolithic northwest China [55]. Haplotype 16129-16223-16298-16327 is found mainly in currently northeast, central and south Siberian populations, in Mongolia and central Asia. It also was found in one ancient Mongolian (2000 yBP) [56]. Haplotype 16093-16129-16223-16298-16311-16327 is probably rare, since it has only been detected previously in four present-day individuals, one in south Siberia, one in Tibet, one in Southeast Asia, and one in China. One Xiaohe individual carried Hg C5 (16223-16288-16298-327), of a variant only observed previously in one individual of southern Siberia, and in one of the Tibetan Plateau (Fig. 3b).

The second most frequent east Eurasian haplogroup in the Xiaohe people was D, found in four individuals, with four different variants. The first, 16051-16223-16362, is found mainly in Southeast Asia. The second, 16223-16234-16316-16362, is found throughout the Eurasian continent, including China, Japan, Siberia, and Eastern Europe. The remaining two D haplotypes had no exact match in any of the available databases. Interestingly, hg D has been observed at high frequency in Hami people, a Bronze Age population of northeast Xinjiang [44]. It is also been observed in Neolithic Chinese and Siberians [43, 55]. In the Network Tree, We can see that some Xiaohe D haplotypes cluster into the East Asian subclade, the others cluster into the Siberian subclade (Fig. 3c). Therefore, the D haplotype sequenced in Xiaohe is currently uninformative about population affinity. One individual carried G2a, but no matching sequence was found in the database. G2a is relatively abundant in northern China and central Asia, reaching significant levels in Southern Siberia [50]. However, Xiaohe G2a haplotype clusters into one of the East Asian clades in the Network tree (Fig. 3d), indicating close affinities to East Asians. One single individual carried hg B, an important East Asian haplogroup, of a particular variant not previously observed. The presence of haplogroups C4, C5, D, G2a and B in Xiaohe people indicates close affinities to Siberians and East Asians.

### Comparison of the Xiaohe population with ancient and extant populations of Eurasia

**Fig. 4:**
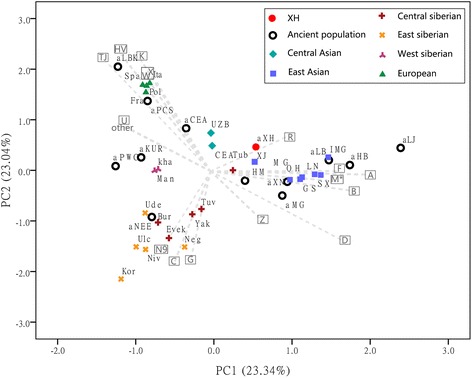
Principal Component Analysis of mitochondrial haplogroup frequencies. The first two dimensions account for 46.38 % of the total variance. Grey arrows represent haplogroup loading vectors, i.e., the contribution of each haplogroup. Ancient populations included in this study: aXH: Xiaohe cemetery; aCA: Nomads from Kazakhstan (2,100–3,400 yBP); aKur: Siberian Kurgans (1,600–3,800 yBP); aPWC: Scandinavian Pitted-Ware Culture foragers (4,500–5,300 yBP); aLBK: German early Neolithic Linear Pottery Culture population(6,900–7,500 yBP);aNEE: North East European ancient people (3,500–7,500 yBP):aLB: Neolithic Lake Baikal population (6,130–7,140 yBP); aHM: Xinjiang Hami people (4000yBP); aHB: Chinese Shanxi Hengbei people (3000yBP); aMG and aLJ: late Neolithic Qijia Culture peopulions in Ganqing region of China (4000yBP); aXN: nomads from Mongolia (2500yBP). Detailed information on the ancient and modern populations is provided in Additional file 3: Table S3

In order to characterise the genetic relationship between the Xiaohe population and other ancient and extant Eurasian populations, the PCA based on the mtDNA haplogroup frequencies and the MDS plot based on genetic distance between sequences were conducted. However, as many individuals had identical C4 haplotypes, indicating potential maternal relationships within the population, the frequency of C4 was likely to be overestimated. To account for this, we assumed a scenario of extreme maternal kinship, where identical haplotypes in several individuals of the same layer were only counted once. The PCA plot of the first two components showed that present-day populations largely segregate into three main clusters: Europeans, Siberians, and Central/East Asians (Fig. 4). Europeans and Central/East Asians were separated along the first component axis (23.34 % of the variance), reflecting their longitude. Europeans and Siberians were separated along the second component axis (23.04 % of the variance). Xiaohe maternal lineages were closest to the Xinjiang populations (modern Xinjiang population and ancient Hami people), and second-closest to the central Siberians (Tuvinians). An MDS plot confirmed the genetic affinity with Siberians inferred from the PCA, but showed a long distance with Central /East Asians (Additional file 7: Figure S2).

## Discussion

Our previous analysis of DNA from the deepest layer of burials of the Xiaohe site revealed that the first settlers had European paternal lineages, and maternal lineages of European and central Siberian origin, consistent with the “steppe hypothesis” of the origins of the first inhabitants of the Tarim Basin [23]. In the present study, analysis of the remaining four, more recent burial layers, confirmed that the origin of the mitochondrial lineages is more widespread, and we detected west Eurasian lineages H, K, U5, U7, U2e, T, east Eurasian lineages B, C4, C5, D, G2a, and Indian lineage M5. Haplotypes H, K, U5 and T are found mostly in Europe, suggesting genetic affinities with Europe. While Xiaohe U2e haplotype has not been observed in living populations, the hg U2e is thought to have originated in Europe, from where it had been spread into central Siberia in the Bronze Age [39]. The distribution of these haplogroups overlaps with the regions of the Afanasievo culture, Andronovo culture or Yamna culture, but is remote from the Oxus civilization. These west Eurasian genetic components in the Xiaohe people corroborate the “steppe hypothesis”.

However, layers 1–4 also had individuals with hgs U7 and M5, common in west/south Asian populations today, but rare in Europeans and Siberians. Although the genetic structure of the oasis people in the Bronze Age is unclear, archaeological evidence indicates that settled populations of the oasis civilization in central Asia descended from farmers from the southwest [17]. These ancient central Asians had been in contact with south Asians and likely received a genetic contribution from them. Considering the archaeological materials and the environmental similarities between central Asia and the Tarim Basin, hgs U7 and M5 observed in Xiaohe people more likely originated from the oasis peoples but not directly from west/south Asians. This suggests populations from the oasis may have made a later contribution to the gene pool of the Xiaohe people, giving some credence to the “oasis hypothesis”. The later Xiaohe people (layers 1–4) carried diverse east Asian maternal lineages, including the predominant C4, as well as C5, which has a similar geographical distribution to C4, suggesting links with Siberia, especially central/south Siberian populations. Although hgs B, D and G2a are common in East Asians and Mongolians besides Siberians, except for broomcorn millet (*P. miliaceum*), there was no archaeological or anthropological evidence in the Xiaohe cemetery for links with East Asia. However, hgs C and D have also been observed in Bronze Age human remains from North Xinjiang (Hami), a place where culture and human features appear to indicate a blend of both east and west. DNA analysis showed that the Hami people had close affinities with Neolithic people in Ganqing region of China [44]. Recently archaeobotanical analysis considered that East Asian domesticated broomcorn likely was introduced into Central Eurasia via the route of North Xinjiang from Ganqing region at middle third millennium BC. Therefore, some eastern components in the later Xiaohe people may have derived from North Xinjiang and have an ultimate origin in East Asia but not central/southern Siberia, something still consistent with the “steppe hypothesis”. This was indicated by the close relationship of the Xiaohe population with populations of Xinjiang in the PCA graph (Fig. 4).

Xiaohe people displays higher and higher levels of haplotype diversity (fifth layer Hd = 0.7381, fourth layer Hd = 0.9004, layers1-3 Hd = 0.9890) from earlier to later, suggesting multiple population incursions into the Tarim Basin after its initial settlement. People carrying European maternal lineages may have spread east into south Siberia, where they mingled with local populations and eventually spread south into Xinjiang via the Ertix River. However, ancient DNA analyses indicate that the west Eurasian lineages observed in ancient south Siberia were associated with the eastward spread of Europeans of the Afanasievo culture [39]. This suggests that the European components could have reached north Xinjiang later, via the Kazakh steppe northwest of the Tarim Basin. Interestingly, the cattle excavated from the Xiaohe cemetery carried mainly lineage T3, typical of European cattle [57]. These diverse lines of evidence support the“steppe hypothesis”. In contrast, people bearing the south /west Asian components could have reached the Tarim Basin through the Pamirs, moving eastward along the south or north edges of the Tarim Basin. Recently one study showed that agricultural populations had contact with nearby mobile pastoralists at the beginning of the second millennium BC in Central Asia [58], indicating that genetic components of agriculturalists might also introgress into pastoralist populations. This was confirmed by the evidence that one Indian haplogroup was found in ancient Kazakhstan [37]. Therefore, people bearing the south/west Asian components could have first married into pastoralist populations, and reached North Xinjiang through the Kazakh steppe following the movement of pastoralist populations, then spread from north Xinjiang southward into the Tarim Basin across the Tianshan Mountains, and intermarried with the earlier inhabitants of the region, giving rise to the later, admixed Xiaohe community. Given that the south/west Asian components are relatively minor in the Xiaohe population, it is likely that nomadic herders from northern steppe had a greater impact on the eastern Tarim Basin than the Central Asian oasis farmers.

The archaeological evidence for woolen textiles and the medicinal plant *Ephedra* in the earliest Xiaohe layer and the Gumugou site indicate that the oasis culture had reached the Tarim Basin in the early Bronze Age. It is well known that *Ephedra* was used by oasis farmers, whereas it does not grow in the Russo-Kazakh steppe, nor is associated with the Afanasievo or Andronovo cultures [5, 7]. Furthermore, the wheat excavated from Xiaohe was hexaploid bread wheat, a cereal grain cultivated originally in the Near East [59]. Therefore, it is possible that the oasis route may have been significant in the peopling of Xinjiang in the early Bronze Age, at least northern or western Xinjiang. This was supported by the evidence that Indian haplogroup M25 was observed in one ancient individual from later Neolithic Ganqing region (data unpublished). The groups reaching the Tarim Basin through the oasis route may have interacted culturally with earlier populations from the steppe, with limited gene flow, resulting in a small genetic signal of the oasis agriculturalists in the Xiaohe community.

## Conclusion

Our data indicate multiple population influences in the Tarim Basin during 4000–3500 yBP, consistent mainly with the “steppe hypothesis”, but with elements of the “oasis hypothesis”. Meanwhile, we can’t exclude the possibility that East Asians had an indirect impact on the Tarim Basin at Bronze Age.

## Additional files

Additional file 1: Table S1. Archaeological information for 92 Xiaohe individuals. Additional file 2: Table S2. Primers used in this study. Additional file 3: Table S3. Ancient and present-day populations used in the principal component analysis. Additional file 4: Table S4. The mtDNA yield of three Xiaohe individuals. Additional file 5: Figure S1. Alignment of cloned mtDNA sequences from six samples. The primer sequences are shadowed. Additional file 6: Table S5. Results of mtDNA HVR-1 multiplex sequencing and the SNP typing. Additional file 7: Figure S2. Multidimensional scaling plot of genetic distances calculated for mtDNA sequences (16050–16391). Population abbreviations are consistent with Fig. 4.

## Abbreviations

PCR: Polymerase chain reaction
mtDNA: Mitochondrial DNA
SNP: Single nucleotide polymorphism
CRS: Cambridge reference sequence
Hg: Haplogroup
MDS: Multidimensional scaling
PCA: Principal component analysis
