# Supplementary material for: Analysis of ancient human mitochondrial DNA from the Xiaohe cemetery: insights into prehistoric population movements in the Tarim Basin, China
Source: BMC Genet. 2015 Jul 8;16:78. doi: 10.1186/s12863-015-0237-5 (PMC4495690; doi:10.1186/s12863-015-0237-5)
Supplement: Additional file 1: Table S1. — Archaeological information for 92 Xiaohe individuals. [file 12863_2015_237_MOESM1_ESM.doc]

**Table S1 Archaeological information for 92 Xiaohe individuals**

| individuals | layer | sex | age | hair color | sampling style |
| --- | --- | --- | --- | --- | --- |
| T18-1 | ? | male | 30-40 |  | femur bone |
| T18-2 | ? | male | 20-30 |  | ulna bone |
| T18-7 | ? | male | — |  | shoulder bone |
| T22-6 | 2 | — | — |  | ulna bone |
| T23-4 | ? | — | adult |  | tibia |
| T24-1 | ? | male | adult |  | bone |
| T24-6 | ? | — | adult |  | bone |
| T24-7 | ? | male | 35-40 |  | shoulder bone |
| T24-12 | ? | male | adult |  | shoulder bone |
| T28-2 | ? | female | 10-11 | Light brown hair | teeth |
| T28-5 | ? | male | — |  | shoulder bone |
| T28-6 | 2 | — | — |  | left crus |
| T28-7 | 2 | — | adult |  | femur |
| T28-8 | ? | male | adult |  | femur |
| T28-9 | ? | — | adult |  | femur |
| T29-5 | ? | male | adult | nut-brown hair | shoulder bone |
| T29-6 | ? | — | adult |  | shoulder bone |
| T29-9 | ? | male | adult |  | shoulder bone |
| T29-12 | ? | male | adult |  | bone |
| T29-14 | 2 | — | adult |  | femur |
| T35-1 | 2 | male | — |  | femur |
| MW | ? | female | — |  | teeth |
| individuals | layer | Sex | Age | Hair color | sampling |
| M12 | 1 | female | adult | flaxen hair | teeth/bone |
| M15 | 1 | female | adult | flaxen hair | teeth/bone |
| M22 | 2 | — | adult |  | shoulder bone |
| M32 | 2 | female | adult | Light brown hair | teeth/femur |
| M35 | 2 | — | adult |  | shoulder bone |
| M39 | 3 | male | adult | Light brown hair | teeth/bone |
| M41 | 3 | female | 35± | Three layer | tibia /femur |
| M55 | 3 | female | — | brown hair | teeth/bone |
| M56 | 3 | male | adult | mid-brown hair | teeth/bone |
| M59 | 3 | male | 40-45 | brown hair | teeth/bone |
| M62 | 3 | male | adult | brown hair | teeth |
| M63 | 3 | male | 30± |  | teeth/bone |
| BM1 | 4 | female | adult |  | teeth/bone |
| BM2 | 4 | female | adult |  | teeth/bone |
| BM5 | 4 | female | 25 | Light brown hair | teeth/bone |
| BM7 | 4 | female | adult |  | tibia |
| BM8 | 4 | male | 20-25 |  | teeth /femur |
| BM9 | 4 | male | 40± |  | teeth/bone |
| BM10 | 4 | male | 35± |  | teeth/bone |
| BM17 | 4 | female | adult | mid-brown hair | teeth/bone |
| BM18 | 4 | female | 50± |  | teeth /Femur |
| BM20 | 4 | female | 25-30 |  | teeth/bone |
| BM22 | 4 | male | 30-35 |  | tibia /femur |
| individuals | layer | Sex | Age | Hair color | sampling |
| BM24 | 4 | male | adult |  | tibia |
| BM25 | 4 | male | adult |  | tibia |
| BM26 | 4 | female | 40-45 | Light brown hair | teeth/bone |
| M70 | 4 | male | 30-40 | brown-black hair | teeth/bone |
| M72 | 4 | female | 9-10 | brown hair | teeth/bone |
| M73 | 4 | male | 14-15 |  | teeth/bone |
| M75 | 4 | female | adult |  | teeth/bone |
| *M84 | 5 | female | adult | black hair(five) | teeth |
| *M85 | 5 | female | adult |  | teeth/bone |
| M87 | 4 | male | 45-50 |  | teeth/bone |
| M88 | 4 | female | adult |  | teeth/bone |
| M89 | 4 | female | 30-35 |  | teeth/bone |
| M93 | 5? | female | 35-40 | mid-brown hair | teeth/bone |
| M92 | 4 | female | adult | Light brown hair | bone |
| M95 | 4 | male | 13-14 | mid-brown hair | bone |
| M97 | 4 | male | adult | brown hair | bone |
| M98 | 4 | female | adult | Light brown hair | teeth/bone |
| M99 | 4 | female | 30-35 | mid-brown hair | bone |
| *M100 | 5 | female | adult |  | teeth/bone |
| *M102 | 5 | female | 25-30 |  | teeth/bone |
| *M104 | 5 | female | 45± | brown-black hair | teeth/bone |
| *M106 | 5 | male | 35**±** |  | teeth/bone |
| *M107 | 5 | female | 40-45 | mid-brown hair | teeth/bone |
| individuals | layer | Sex | Age | Hair color | sampling |
| *M108 | 5 | female | — |  | teeth/bone |
| *M109 | 5 | female | 50**±** | Light brown hair | teeth/bone |
| *M110 | 5 | female | 19-20 | brown hair | teeth/bone |
| *M111 | 5 | male | 25-30 | brown hair | teeth/bone |
| *M112 | 5 | female | 25-30 | brown hair | teeth/bone |
| *M114 | 5 | male | — |  | teeth/bone |
| *M115 | 5 | male | 35-40 | Brown black hair | teeth/bone |
| *M117 | 5 | female | 40± |  | teeth/bone |
| *M119 | 5 | female | 30-35 | Light brown hair | teeth/bone |
| *M120 | 5 | male | 45**±** | Brown black hair | teeth/bone |
| *M121 | 5 | male | adult |  | teeth |
| *M125 | 5 | female | adult | flaxen hair | teeth/bone |
| *M127 | 5 | female | 55**±** |  | teeth/bone |
| *M128 | 5 | female | 40± | Brown black hair | teeth/bone |
| M129 | 4 | male | 45-50 |  | teeth/bone |
| M130 | 4 | male | 25-30 |  | teeth/bone |
| *M131 | 5 | female | 30-35 | mid-brown hair | teeth/bone |
| *M132 | 5 | female | 40**±** | mid-brown hair | teeth/bone |
| *M134 | 5 | — | 14-15 | Brown black hair | teeth/bone |
| *M135 | 5 | female | 50**±** | black hair | teeth/bone |
| *M136 | 5 | male | 35**±** | mid-brown hair | teeth/bone |
| *M137 | 5 | female | 45-50 |  | teeth/bone |
| *M138 | 5 | female | 35± | Brown black hair | teeth/bone |
| individuals | layer | Sex | Age | Hair color | sampling |
| *M139 | 5 | male | 40-45 | Brown hair | teeth/bone |

Note: Tombs from layers1-2 were destroyed by looters and weather, and 22 samples (shown as T) were chose and represented 22 individuals. An asterisk (*) identifies samples analysed in a previous study by Li *et al.* (2010). A hyphen (-) and blank means inconclusive results; a question mark (?) means the sample is from the first or second layer. In this analysis, we pooled the three upper layers into one group, called upper layer.
