# Supplementary material for: Analysis of ancient human mitochondrial DNA from the Xiaohe cemetery: insights into prehistoric population movements in the Tarim Basin, China
Source: BMC Genet. 2015 Jul 8;16:78. doi: 10.1186/s12863-015-0237-5 (PMC4495690; doi:10.1186/s12863-015-0237-5)
Supplement: Additional file 2: Table S2. — Primers used in this study. [file 12863_2015_237_MOESM2_ESM.doc]

| Haplogroup/AMG | Primer | SNP | Length |
| --- | --- | --- | --- |
| HVRI-AB | L16017 5'-TTCTCTGTTCTTTCATGGGGA  H16251 5'-GGAGTTGCAGTTGATGTGTGA | Sequencing | 235bp |
| HVRI-CD | L16201 5'-CAAGCAAGTACAGCAATCAAC  H16409 5'-AGGATGGTGGTCAAGGGA | Sequencing | 209bp |
| M | 10400T 5'-taattaTACAAAAAGGATTAGACTGtgCT  10400C 5'-TACAAAAAGGATTAGACaGAACC  10400R 5'-GAAGTGAGATGGTAAATGCTAG | 10400T | 149bp/142bp |
| R | L12604 5'-ATCCCTGTAGCATTGTTCG  H12754 5'-GTTGGAATAGGTTGTTAGCG | 12705C | 151bp |
| UK | L12247 5‘-TAACAACATGGCTTTCTCAACT H12377 5‘-GAAGTCAGGGTTAGGGTGGT | 12308G | 132bp |
| C | L14318T 5'-CCTTCATAAATTATTCAGCTTCCaACACTAT  L14318C 5'-aaaaagctaCATAAATTATTCAGCTTCCTACtCTAC  H14318R 5'-TTAGTGGGGTTAGCGATGGA | 14318C | 110bp/115bp |
| C4 | L11845 5'- AAGCCTCGCTAACCTCGCC  H12120 5'- GGGTGAGTGAGCCCCATTG | 11969 | 176bp |
| B | L8215 5' ACAGTTTCATGCCCATCGTC '  H8297 5' ATGCTAAGTTAGCTTTACAG | CoII/tRNAlys  9-bp deletion | 121bp/112bp |
| D | 5178A 5' TGATCAACGCACCTGAAACAAGA 3'  5178C 5' GTCGCACCTGAAGCAAGC 3'  5178R 5' CCCATTTGAGCAAAAAGCC 3' | 5178A | 107/102bp |
| G | L4735 5’TTACCTCCTCAAAGCAATACA3’  H4899 5’GGTATATGATTGAGATGGG3’ | 4833G | 165bp |
| M5 | L1812 5'-CCAAGCATAATATAGCAAGG  H1964 5'- ATTTTGCTACATAGACGGGT | 1888A | 153bp |
| M25 | L15871 5'-AATACTCAAATGGGCCTGTCC  H16039 5'-CTTCCCCATGAAAGAACAGAGA | 15928A | 169bp |
| R1 | L4812 5'- GTCCCAGAGGTTACCCAAG  H4975 5'- CCACCTCAACTGCCTGCTA | 4917G | 164bp |
| R11 | L9920 5'- CGCCTGATACTGGCATTTTGT  H101075' -GTAGTAAGGCTAGGAGGGTGTTG | 10031C | 188bp |
| HV | L14668 5'- CATCATTATTCTCGCACGG  H14831 5'- CGGAGATGTTGGATGGGGT | 14766C | 164bp |
| H | L6966 5‘-GGCATTGTATTAGCAAACTCAT H7118 5‘-TAGGGTGTAGCCTGAGAATAG | 7028C | 152bp |
| T | L15589 5'-CCGATCCGTCCCTAACAAG  L15584 5'-TAACTCCTATCCGTCCCTAACAAA  H15713 5'-ATTGGCTTAGTGGGCGAAA | 15607G | 125bp/130bp |
| AMG | AMG1 5'-CCTGGGCTCTGTAAAGAATAG  AMG2 5'-CAGAGCTTAAACTGGGAAGCTG |  | 115bp/121bp |
