# Supplementary material for: Analysis of ancient human mitochondrial DNA from the Xiaohe cemetery: insights into prehistoric population movements in the Tarim Basin, China
Source: BMC Genet. 2015 Jul 8;16:78. doi: 10.1186/s12863-015-0237-5 (PMC4495690; doi:10.1186/s12863-015-0237-5)
Supplement: Additional file 3: Table S3. — Ancient and present-day populations used in the principal component analysis. [file 12863_2015_237_MOESM3_ESM.doc]

| population names | label | n | references | position |
| --- | --- | --- | --- | --- |
| **modern populations** | | | | |
| Mongolians | MN | 103 | 1 | Mongolia |
| Inner Mongolians | IMN | 155 | 1 | North China |
| Gansu | GS | 128 | 2 |
| Qinghai | QH | 186 | 2 |
| Xinjiang | XJ | 457 | 3 |
| Shaanxi | SX | 138 | 2 |
| Liaoning | LN | 102 | 2 |
| Yakuts | Yak | 191 | 4 | Central Siberia |
| Tuvinians | Tuv | 131 | 4,5 |
| Evenks | Eve | 71 | 5 |
| Buyats | Buy | 25 | 5 |
| Tubalars | Tub | 72 | 5 |
| Ulchi | Ulc | 87 | 5 | East Siberia |
| Koriar | Kor | 155 | 4 |
| Negidal | Neg | 33 | 5 |
| Udegey | Ude | 46 | 5 |
| Nivkhi | Niv | 56 | 5 |
| Mansi | Man | 161 | 5,6 | West Siberia |
| Khanty | Kha | 106 | 6 |
| Poland | Pol | 436 | 7 | Europe |
| France | Fra | 210 | 8 |
| Italy | Ita | 865 | 9 |
| Spain | Spa | 279 | 10 |
| Uzbekistan | UZB | 1575 | 11 | Central Asia |
| Central Asian | CEA | 437 | 3 |
| **ancient populations** | | | | |
| South Siberian Kurgan | aKur | 26 | 12 | Central Siberia |
| Neolithic Lake Baikal | aLB | 30 | 13 | Central Siberia |
| nomads of the Xiongnu | aXN | 46 | 14 | Mongolia |
| Xinjiang Hami | aHM | 24 | 15 | western China |
| Shanxi Hengbei | aHB | 64 | In press | Central China |
| Xinjiang Xiaohe | aXH | 56 | This study | western China |
| Ganqing Megou | aMG | 55 | unpublished | western China |
| Ganqing Lajia | aLJ | 27 | unpublished | western China |
| nomads of central Asian | aCEA | 27 | 16 | Central Asia |
| Scandinavian Pitted-Ware Culture foragers | aPWC | 19 | 17 | North Europe |
| Eneolithic/ Bronze Age individuals from the North Pontic steppe | aCPS | 62 | 18 | East Europe |
| North East European ancient individuals | aNEE | 34 | 19 | northwest Russia |
| early Neolithic Linear Pottery Culture foragers | aLBK | 42 | 20 | Central Europe |

**Table S3. Details of ancient and modern populations used in PCA and MDS.**

1. [Cheng B](http://www.ncbi.nlm.nih.gov/pubmed?term=Cheng B[Author]&cauthor=true&cauthor_uid=18769869), [Tang W](http://www.ncbi.nlm.nih.gov/pubmed?term=Tang W[Author]&cauthor=true&cauthor_uid=18769869), [He L](http://www.ncbi.nlm.nih.gov/pubmed?term=He L[Author]&cauthor=true&cauthor_uid=18769869), [Dong Y](http://www.ncbi.nlm.nih.gov/pubmed?term=Dong Y[Author]&cauthor=true&cauthor_uid=18769869), [Lu J](http://www.ncbi.nlm.nih.gov/pubmed?term=Lu J[Author]&cauthor=true&cauthor_uid=18769869), [Lei Y](http://www.ncbi.nlm.nih.gov/pubmed?term=Lei Y[Author]&cauthor=true&cauthor_uid=18769869), [Yu H](http://www.ncbi.nlm.nih.gov/pubmed?term=Yu H[Author]&cauthor=true&cauthor_uid=18769869), [Zhang J](http://www.ncbi.nlm.nih.gov/pubmed?term=Zhang J[Author]&cauthor=true&cauthor_uid=18769869), [Xiao C](http://www.ncbi.nlm.nih.gov/pubmed?term=Xiao C[Author]&cauthor=true&cauthor_uid=18769869): **Genetic imprint of the Mongol: signal from phylogeographic analysis of mitochondrial DNA**.[*J Hum Genet*](http://www.ncbi.nlm.nih.gov/pubmed/18769869)2008*,* **53:**905-913. (doi: 10.1007/s10038-008-0325-8)
2. Zhao M, Kong QP, [Wang HW](http://www.ncbi.nlm.nih.gov/pubmed?term=Wang HW[Author]&cauthor=true&cauthor_uid=19955425), [Peng MS](http://www.ncbi.nlm.nih.gov/pubmed?term=Peng MS[Author]&cauthor=true&cauthor_uid=19955425), [Xie XD](http://www.ncbi.nlm.nih.gov/pubmed?term=Xie XD[Author]&cauthor=true&cauthor_uid=19955425), [Wang WZ](http://www.ncbi.nlm.nih.gov/pubmed?term=Wang WZ[Author]&cauthor=true&cauthor_uid=19955425), [Jiayang](http://www.ncbi.nlm.nih.gov/pubmed?term=Jiayang[Author]&cauthor=true&cauthor_uid=19955425), [Duan JG](http://www.ncbi.nlm.nih.gov/pubmed?term=Duan JG[Author]&cauthor=true&cauthor_uid=19955425), [Cai MC](http://www.ncbi.nlm.nih.gov/pubmed?term=Cai MC[Author]&cauthor=true&cauthor_uid=19955425), [Zhao SN](http://www.ncbi.nlm.nih.gov/pubmed?term=Zhao SN[Author]&cauthor=true&cauthor_uid=19955425), *et al*: **Mitochondrial genome evidence reveals successful Late Paleolithic settlement on the Tibetan Plateau**. *Proc Natl Acad Sci* 2009, **106**:21230-21235. (doi: 10.1073/pnas.0907844106)
3. Yao YG, Kong QP, Wang CY, Zhu CL, Zhang YP: **Different matrilineal contributions to genetic structure of ethnic groups in the Silk Road region in China**. *Mol Biol Evol* 2004, **21**:2265-2280. (doi:10.1093/molbev/msh238).
4. Fedorova SA, Bermisheva MA, Villems R, Maksimova NR, Khusnutdinova EK:[**Analysis of mitochondrial DNA haplotypes in Yakut population**.](http://www.ncbi.nlm.nih.gov/pubmed/12942638)*Mol Biol (Mosk)* 2003, **37**: 643-653.
5. Starikovskaya EB, Sukernik RI, Derbeneva OA, Volodko NV, Ruiz-Pesini E, Torroni A, Brown MD, Lott MT, Hosseini SH, Huoponen K*, et al:* **Mitochondrial DNA diversity in indigenous populations of the southern extent of Siberia, and the origins of Native American haplogroups**. *Ann Hum Genet* 2005, **69**:67-89. (doi:10.1046/j.1529-8817.2003.00127.x)
6. [Pimenoff VN](http://www.ncbi.nlm.nih.gov/pubmed/?term=Pimenoff VN%5BAuthor%5D&cauthor=true&cauthor_uid=18506205), [Comas D](http://www.ncbi.nlm.nih.gov/pubmed/?term=Comas D%5BAuthor%5D&cauthor=true&cauthor_uid=18506205), [Palo JU](http://www.ncbi.nlm.nih.gov/pubmed/?term=Palo JU%5BAuthor%5D&cauthor=true&cauthor_uid=18506205), [Vershubsky G](http://www.ncbi.nlm.nih.gov/pubmed/?term=Vershubsky G%5BAuthor%5D&cauthor=true&cauthor_uid=18506205), [Kozlov A](http://www.ncbi.nlm.nih.gov/pubmed/?term=Kozlov A%5BAuthor%5D&cauthor=true&cauthor_uid=18506205), [Sajantila A](http://www.ncbi.nlm.nih.gov/pubmed/?term=Sajantila A%5BAuthor%5D&cauthor=true&cauthor_uid=18506205): **Northwest Siberian Khanty and Mansi in the junction of West and East Eurasian gene pools as revealed by uniparental markers.** [*Eur J Hum Genet*](http://www.ncbi.nlm.nih.gov/pubmed/?term=Northwest+Siberian+Khanty+and+Mansi+in+the+junction+of+West+and+East+Eurasian+gene+pools+as+revealed+by+uniparental+markers)2008*,***16**(10):1254-1264. (doi: 10.1038/ejhg.2008.101)
7. Malyarchuk BA, Grzybowski T, Derenko MV, Czarny J, Wozniak M, et al: **Mitochondrial DNA variability in Poles and Russians**. *Ann Hum Genet* 2002, **66**:261-283.
8. Dubut V, Chollet L, Murail P, Cartault F, Beraud-Colomb E, Serre M, Mogentale-Profizi N: **mtDNA polymorphisms in five French groups: importance of regional sampling**. *Eur J Hum Genet* 2004, **12**: 293-300. (doi:10.1038/sj.ejhg.5201145).
9. [Boattini A](http://www.ncbi.nlm.nih.gov/pubmed/?term=Boattini A%5BAuthor%5D&cauthor=true&cauthor_uid=23734255), [Martinez-Cruz B](http://www.ncbi.nlm.nih.gov/pubmed/?term=Martinez-Cruz B%5BAuthor%5D&cauthor=true&cauthor_uid=23734255), [Sarno S](http://www.ncbi.nlm.nih.gov/pubmed/?term=Sarno S%5BAuthor%5D&cauthor=true&cauthor_uid=23734255), [Harmant C](http://www.ncbi.nlm.nih.gov/pubmed/?term=Harmant C%5BAuthor%5D&cauthor=true&cauthor_uid=23734255), [Useli A](http://www.ncbi.nlm.nih.gov/pubmed/?term=Useli A%5BAuthor%5D&cauthor=true&cauthor_uid=23734255), [Sanz P](http://www.ncbi.nlm.nih.gov/pubmed/?term=Sanz P%5BAuthor%5D&cauthor=true&cauthor_uid=23734255), [Yang-Yao D](http://www.ncbi.nlm.nih.gov/pubmed/?term=Yang-Yao D%5BAuthor%5D&cauthor=true&cauthor_uid=23734255), [Manry J](http://www.ncbi.nlm.nih.gov/pubmed/?term=Manry J%5BAuthor%5D&cauthor=true&cauthor_uid=23734255), [Ciani G](http://www.ncbi.nlm.nih.gov/pubmed/?term=Ciani G%5BAuthor%5D&cauthor=true&cauthor_uid=23734255), [Luiselli D](http://www.ncbi.nlm.nih.gov/pubmed/?term=Luiselli D%5BAuthor%5D&cauthor=true&cauthor_uid=23734255), et al: **Uniparental markers in Italy reveal a sex-biased genetic structure and different historical strata.** [*PLoS One* 2013,](http://www.ncbi.nlm.nih.gov/pubmed/23734255) **8**(5):e65441. (doi: 10.1371/journal.pone.0065441)
10. [Hernández CL](http://www.ncbi.nlm.nih.gov/pubmed/?term=Hernández CL%5BAuthor%5D&cauthor=true&cauthor_uid=24460736), [Reales G](http://www.ncbi.nlm.nih.gov/pubmed/?term=Reales G%5BAuthor%5D&cauthor=true&cauthor_uid=24460736), [Dugoujon JM](http://www.ncbi.nlm.nih.gov/pubmed/?term=Dugoujon JM%5BAuthor%5D&cauthor=true&cauthor_uid=24460736), [Novelletto A](http://www.ncbi.nlm.nih.gov/pubmed/?term=Novelletto A%5BAuthor%5D&cauthor=true&cauthor_uid=24460736), [Rodríguez JN](http://www.ncbi.nlm.nih.gov/pubmed/?term=Rodríguez JN%5BAuthor%5D&cauthor=true&cauthor_uid=24460736), [Cuesta P](http://www.ncbi.nlm.nih.gov/pubmed/?term=Cuesta P%5BAuthor%5D&cauthor=true&cauthor_uid=24460736), [Calderón R](http://www.ncbi.nlm.nih.gov/pubmed/?term=Calderón R%5BAuthor%5D&cauthor=true&cauthor_uid=24460736): **Human maternal heritage in Andalusia (Spain): its composition reveals high internal complexity and distinctive influences of mtDNA haplogroups U6 and L in the western and eastern side of region.** [*BMC Genet*](http://www.ncbi.nlm.nih.gov/pubmed/24460736)2014*,***15**:11. (doi: 10.1186/1471-2156-15-11)
11. [Irwin JA](http://www.ncbi.nlm.nih.gov/pubmed?term=Irwin JA[Author]&cauthor=true&cauthor_uid=20140442), [Ikramov A](http://www.ncbi.nlm.nih.gov/pubmed?term=Ikramov A[Author]&cauthor=true&cauthor_uid=20140442), [Saunier J](http://www.ncbi.nlm.nih.gov/pubmed?term=Saunier J[Author]&cauthor=true&cauthor_uid=20140442), [Bodner M](http://www.ncbi.nlm.nih.gov/pubmed?term=Bodner M[Author]&cauthor=true&cauthor_uid=20140442), [Amory S](http://www.ncbi.nlm.nih.gov/pubmed?term=Amory S[Author]&cauthor=true&cauthor_uid=20140442), [Röck A](http://www.ncbi.nlm.nih.gov/pubmed?term=R?ck A[Author]&cauthor=true&cauthor_uid=20140442), [O'Callaghan J](http://www.ncbi.nlm.nih.gov/pubmed?term=O'Callaghan J[Author]&cauthor=true&cauthor_uid=20140442), [Nuritdinov A](http://www.ncbi.nlm.nih.gov/pubmed?term=Nuritdinov A[Author]&cauthor=true&cauthor_uid=20140442), [Atakhodjaev S](http://www.ncbi.nlm.nih.gov/pubmed?term=Atakhodjaev S[Author]&cauthor=true&cauthor_uid=20140442), [Mukhamedov R](http://www.ncbi.nlm.nih.gov/pubmed?term=Mukhamedov R[Author]&cauthor=true&cauthor_uid=20140442),et al: **The mtDNA composition of Uzbekistan: a microcosm of Central Asian patterns**.[*Int J Legal Med*](http://www.ncbi.nlm.nih.gov/pubmed/20140442) **2010, 124**:195-204. (doi: 10.1007/s00414-009-0406-z)
12. Keyser C, Bouakaze C, Crubezy E, Nikolaev VG, Montagnon D, Reis T, Ludes B: **Ancient DNA provides new insights into the history of south Siberian Kurgan people**. *Hum Genet* 2009, **126**:395-410. (doi:10.1007/s00439-009-0683-0).
13. Mooder KP, Weber AW, Bamforth FJ, Lieverse AR, Schurr TG, Bazaliiski VI, Savel'ev NA: **Matrilineal affinitiesand prehistoric Siberian mortuary practices: a case study from Neolithic Lake Baikal**. *J Archaeol Sci* 2005, **32**: 619-634. (doi:10.1016/j.jas.2004.12.002)
14. Keyser-Tracqui C, Crubézy E, Ludes B: **Nuclear and mitochondrial DNA analysis of a 2,000- year-old necropolis in the Egyin Gol Valley of Mongolia**. *Am J Hum Genet* 2003, **73**:247-260.
15. [Gao SZ](http://www.ncbi.nlm.nih.gov/pubmed/?term=Gao SZ%5BAuthor%5D&cauthor=true&cauthor_uid=25546319), [Zhang Y](http://www.ncbi.nlm.nih.gov/pubmed/?term=Zhang Y%5BAuthor%5D&cauthor=true&cauthor_uid=25546319), [Wei D](http://www.ncbi.nlm.nih.gov/pubmed/?term=Wei D%5BAuthor%5D&cauthor=true&cauthor_uid=25546319), [Li HJ](http://www.ncbi.nlm.nih.gov/pubmed/?term=Li HJ%5BAuthor%5D&cauthor=true&cauthor_uid=25546319), [Zhao YB](http://www.ncbi.nlm.nih.gov/pubmed/?term=Zhao YB%5BAuthor%5D&cauthor=true&cauthor_uid=25546319), [Cui YQ](http://www.ncbi.nlm.nih.gov/pubmed/?term=Cui YQ%5BAuthor%5D&cauthor=true&cauthor_uid=25546319), [Zhou H](http://www.ncbi.nlm.nih.gov/pubmed/?term=Zhou H%5BAuthor%5D&cauthor=true&cauthor_uid=25546319). **Ancient DNA reveals a migration of the ancient Di-qiang populations into Xinjiang as early as the early Bronze Age .**[*Am J Phys Anthropol*](http://www.ncbi.nlm.nih.gov/pubmed/25546319)2014, published online (doi: 10.1002/ajpa.22690)
16. Lalueza-Fox C, Sampietro ML, Gilbert MT, Castri L, Facchini F, Pettener D, Bertranpetit J: **Unravelling migrations in the steppe: mitochondrial DNA sequences from ancient central Asians**. *Proc Biol Sci* 2004, **271**:941-947. (doi:10.1098/rspb.2004.2698).
17. Malmstrom H, Gilbert MT, Thomas MG, Brandstrom M, Stora J, Molnar P, Andersen PK, Bendixen C, Holmlund G, Gotherstrom A*, et al:***Ancient DNA reveals lack of continuity between neolithic hunter-gatherers and contemporary Scandinavians**. *Curr Biol* 2009*,* **19**:1758-1762. (doi:10.1016/j.cub.2009.09.017).
18. [Wilde S](http://www.ncbi.nlm.nih.gov/pubmed?term=Wilde S[Author]&cauthor=true&cauthor_uid=24616518), [Timpson A](http://www.ncbi.nlm.nih.gov/pubmed?term=Timpson A[Author]&cauthor=true&cauthor_uid=24616518), [Kirsanow K](http://www.ncbi.nlm.nih.gov/pubmed?term=Kirsanow K[Author]&cauthor=true&cauthor_uid=24616518), [Kaiser E](http://www.ncbi.nlm.nih.gov/pubmed?term=Kaiser E[Author]&cauthor=true&cauthor_uid=24616518), [Kayser M](http://www.ncbi.nlm.nih.gov/pubmed?term=Kayser M[Author]&cauthor=true&cauthor_uid=24616518), [Unterländer M](http://www.ncbi.nlm.nih.gov/pubmed?term=Unterländer M[Author]&cauthor=true&cauthor_uid=24616518), [Hollfelder N](http://www.ncbi.nlm.nih.gov/pubmed?term=Hollfelder N[Author]&cauthor=true&cauthor_uid=24616518), [Potekhina ID](http://www.ncbi.nlm.nih.gov/pubmed?term=Potekhina ID[Author]&cauthor=true&cauthor_uid=24616518), [Schier W](http://www.ncbi.nlm.nih.gov/pubmed?term=Schier W[Author]&cauthor=true&cauthor_uid=24616518), [Thomas MG](http://www.ncbi.nlm.nih.gov/pubmed?term=Thomas MG[Author]&cauthor=true&cauthor_uid=24616518), [Burger J](http://www.ncbi.nlm.nih.gov/pubmed?term=Burger J[Author]&cauthor=true&cauthor_uid=24616518): **Direct evidence for positive selection of skin, hair, and eye pigmentation in Europeans during the last 5,000 y**. [*Proc Natl Acad Sci*](http://www.ncbi.nlm.nih.gov/pubmed/?term=ancient+DNA++Wilde) 2014, **111(13)**:4832-4837. (doi: 10.1073/pnas.1316513111)
19. Sarkissian CD, Balanovsky O, Brandt G, Khartanovich V, Buzhilova A, Koshel S, Zaporozhchenko V, Gronenborn D, Moiseyev V, Kolpakov E, et al. **Ancient DNA reveals prehistoric gene-flow from Siberia in the complex human population history of North East Europe**. *PLoS Genet* **2013, 9**: e1003296. (doi:10.1371/journal.pgen.1003296).
20. [Haak W](http://www.ncbi.nlm.nih.gov/pubmed/?term=Haak W%5BAuthor%5D&cauthor=true&cauthor_uid=21085689), [Balanovsky O](http://www.ncbi.nlm.nih.gov/pubmed/?term=Balanovsky O%5BAuthor%5D&cauthor=true&cauthor_uid=21085689), [Sanchez JJ](http://www.ncbi.nlm.nih.gov/pubmed/?term=Sanchez JJ%5BAuthor%5D&cauthor=true&cauthor_uid=21085689), [Koshel S](http://www.ncbi.nlm.nih.gov/pubmed/?term=Koshel S%5BAuthor%5D&cauthor=true&cauthor_uid=21085689), [Zaporozhchenko V](http://www.ncbi.nlm.nih.gov/pubmed/?term=Zaporozhchenko V%5BAuthor%5D&cauthor=true&cauthor_uid=21085689), [Adler CJ](http://www.ncbi.nlm.nih.gov/pubmed/?term=Adler CJ%5BAuthor%5D&cauthor=true&cauthor_uid=21085689), [Der Sarkissian CS](http://www.ncbi.nlm.nih.gov/pubmed/?term=Der Sarkissian CS%5BAuthor%5D&cauthor=true&cauthor_uid=21085689), [Brandt G](http://www.ncbi.nlm.nih.gov/pubmed/?term=Brandt G%5BAuthor%5D&cauthor=true&cauthor_uid=21085689), [Schwarz C](http://www.ncbi.nlm.nih.gov/pubmed/?term=Schwarz C%5BAuthor%5D&cauthor=true&cauthor_uid=21085689), [Nicklisch N](http://www.ncbi.nlm.nih.gov/pubmed/?term=Nicklisch N%5BAuthor%5D&cauthor=true&cauthor_uid=21085689), et al. **Ancient DNA from European early neolithic farmers reveals their near eastern affinities**. [*PLoS Biol*](http://www.ncbi.nlm.nih.gov/pubmed/?term=Ancient+DNA+from+European+Early+Neolithic+Farmers+Reveals+Their+Near+Eastern+Affinities) **2010, 8**(11):e1000536. (doi: 10.1371/journal.pbio.1000536)
