# Supplementary material for: Analysis of ancient human mitochondrial DNA from the Xiaohe cemetery: insights into prehistoric population movements in the Tarim Basin, China
Source: BMC Genet. 2015 Jul 8;16:78. doi: 10.1186/s12863-015-0237-5 (PMC4495690; doi:10.1186/s12863-015-0237-5)
Supplement: Additional file 4: Table S4. — The mtDNA yield of three Xiaohe individuals. [file 12863_2015_237_MOESM4_ESM.doc]

**Table S4 .**The mtDNA yield of three Xiaohe individuals

| samples | yield (copies/uL) | | | |
| --- | --- | --- | --- | --- |
|  | 138bp | 209 bp | 238 bp | 363 bp |
| BM1  BM9  M130  Negative  Control DNA | 250-750  700-750  1000-1200  -  1.53E-15 | 100-150  400-550  500-750  -  1.26E-15 | 80-100  300-500  350-500  -  1.2E-15 | -  -  <30  -  1.92E-15 |
