# Supplementary material for: Analysis of ancient human mitochondrial DNA from the Xiaohe cemetery: insights into prehistoric population movements in the Tarim Basin, China
Source: BMC Genet. 2015 Jul 8;16:78. doi: 10.1186/s12863-015-0237-5 (PMC4495690; doi:10.1186/s12863-015-0237-5)
Supplement: Additional file 5: Figure S1. — Alignment of cloned mtDNA sequences from six samples. The primer sequences are shadowed. [file 12863_2015_237_MOESM5_ESM.pdf]

```
         16017                                                                                                                                                                                                                        16251
         ↓                                                                                                                                                                                                                            ↓
CRS      TTCT CTGTTCTTTC ATGGGGAAGC AGATTTGGGT ACCACCCAAG TATTGACTCA CCCATCAACA ACCGCTATGT ATTTCGTACA TTACTGCCAG CCACCATGAA TATTGTACGG TACCATAAAT ACTTGACCAC CTGTAGTACA TAAAAACCCA ATCCACATCA AAACCCCCTC CCCATGCTTA CAAGCAAGTA CAGCAATCAA CCCTCAACTA TCACACATCA ACTGCAACTC C
T23-4AB1 .... .......... .......... .......... .....T.... .......... .......... .......... .......... .......... .......... .......... .......... .......... .......... .......... .......... .......... .......... .......... .......... .......... .......... .......... .
T23-4AB2 .... .......... .......... .......... .......... .......... .......... .......... .......... ......T... .......... .......... .......... .......... .......... .......... .......... .......... .......... .......... .......... .......... .......... .......... .
T23-4AB3 .... .......... .......... .......... .......... .......... .......... .......... .......... .......... .......... .......... .......... .......... .......... .......... .......... .......... .......... .......... .........G .......... .......... .......... .
T23-4AB4 .... .......... .......... .......... .......... .......... .......... .......... .......... .......... .......... .......... .......... .......... .......... .......... .......... .......... .......... .......... .......... .......... .......... .......... .
T23-4AB5 .... .......... .......... .......... .......... .......... .......... .......... .......... .......... .......... .......... .......... .......... .......... .......... .......... .......... .......... .......... .......... .......... .......... .......... .
T23-4AB6 .... .......... .......... .......... .......... .......... .......... .......... .......... .......... .......... .......... .......... .......... .......... .......... .......... .......... .......... .......... .......... .......... .......... .......... .
T23-4AB7 .... .......... .......... .......... .......... .......... .......... .......... .......... .......... .......... .......... .......... .......... .......... .......... .......... .......... .......... .......... .......... .......... .......... .......... .
T23-4AB8 .... .......... .......... .......... .......... .......... .......... .......... .......... .......... .......... .......... .......... .......... .......... .......... .......... .......... .......... .......... .......... .......... .......... .......... .
T28-8AB1 .... .......... .......... .......... .......... .......... .......... .......... .......... .......... .......... .......... .......... .......... .......... .......... .......... .CC.....C. .......... .......... ......C... .......... .......... .......... .
T28-8AB2 .... .......... .......... .......... .......... .......... .......... .......... .......... .......... .......... .......... .......... .......... .......... .......... .......... .CC.....C. .......... .......... ......C... .......... .......... .......... .
T28-8AB3 .... .......... .......... .......... .......... .......... .......... .......... .......... .......... .......... .......... .......... .......... .......... .......... .......... .CC.....C. .......... .......... ......C... .......... .......... .......... .
T28-8AB4 .... .......... .......... .......... .......... .......... .......... .......... .......... .......... .......... .......... .......... .......... .......... .......... .......... .CC.....C. .......... .......... ......C... .......... .......... .......... .
T28-8AB5 .... .......... .......... .......... .......... .......... .......... .......... .......... .......... .......... .......... .......... .......... .......... .......... .......... .CC.....C. .......... .......... ......C... .......... .......... .......... .
T28-8AB6 .... .......... .......... .......... .......... .......... .......... .......... .......... .......... .......... .......... .......... .......... .......... .......... .......... .CC.....C. .......... .......... ......C... .......... .......... .......... .
T28-8AB7 .... .......... .......... .......... .......... .......... .......... .......... .......... .......... .......... .......... .......... .......... .......... .......... .......... .CC.....C. .......... .......... ......C... .......... .......... .......... .
T28-8AB8 .... .......... .......... .......... .......... .......... .......... .......... .......... .......... .......... .......... .......... .......... .......... .......... .......... .CC.....C. .......... .......... ......C... .......... .......... .......... .
T28-5AB1 .... .......... .......... .......... .......... .......... .......... .......... .......... .......... .......... .......... .......... .......... .......... .......... .......... .......... .T........ .......... .......... .......... .......... .......... .
T28-5AB2 .... .......... .......... .......... .......... .......... .......... .......... .......... .......... .......... .......... .......... .......... .......... .......... .......... .......... .T........ .......... .......... .......... .......... .......... .
T28-5AB3 .... .......... .......... .......... .......... .......... .......... .......... .......... .......... .......... .......... .......... .......... .......... .......... .......... .......... .T........ .......... .......... .......... .......... .......... .
T28-5AB4 .... .......... .......... .......... .......... .......... .......... .......... .......... .......... .......... .......... .......... .......... .......... .......... .......... .......... .T........ .......... .......... .......... .......... .......... .
T28-5AB5 .... .......... .......... .......... .......... .......... .......... .......... .......... .......... .......... .......... .......... .......... .......... .......... .......... .......... .T........ .......... .......... .......... .......... .......... .
T28-5AB6 .... .......... .......... .......... .......... .......... .......... .......... .......... .......... .......... .......... .......... .......... .......... .......... .......... .......... .T........ .......... .......... .......... .......... .......... .
T28-5AB7 .... .......... .......... .......... .......... .......... .......... .......... .......... .......... .......... .......... .......... .......... .......... .......... .......... .......... .T........ .......... .......... .......... .......... .......... .
T28-5AB8 .... .......... .......... .......... .......... .......... .......... .......... .......... .......... .......... .......... .......... .......... .......... .G........ .......... .......... .......... .......... .......... ..T....... .......... .......... .

         16201                                                                                                                                                                                    16409
         ↓                                                                                                                                                                                        ↓
CRS      CAAGCAAGTA CAGCAATCAA CCCTCAACTA TCACACATCA ACTGCAACTC CAAAGCCACC CCTCACCCAC TAGGATACCA ACAAACCTAC CCACCCTTAA CAGTACATAG TACATAAAGC CATTTACCGT ACATAGCACA TTACAGTCAA ATCCCTTCTC GTCCCCATGG ATGACCCCCC TCAGATAGGG GTCCCTTGAC CACCATCCT
T18-1CD1 .......... .......... ..T....... .......... .......... .......... .......... .......... .......... .......... .......... .......... .......... .......... .......... .......... .C........ .......... .......... .......... .........
T18-1CD2 .......... .......... ..T....... .......... .......... .......... .......... .......... .......... .......... .......... .......... .......... .......... .......... .......... .C........ .......... .......... .......... .........
T18-1CD3 .......... .......... ..T....... .......... .......... .......... .......... .......... .......... .......... .......... .......... .......... .......... .......... .......... .C........ .......... .......... .......... .........
T18-1CD4 .......... .......... ..T....... .......... .......... .......... .......... .......... .......... .......... .......... .......... .......... .......... .......... .......... .C........ .......... .......... .......... .........
T18-1CD5 .......... .......... ..T....... .......... .......... .......... .......... .......... .......... .......... .......... .......... .......... .......... .......... .......... .C........ .......... .......... .......... .........
T18-1CD6 .......... .......... ..T....... .......... .......... .......... .......... .......... .......... .......... .......... .......... .......... .......... .......... .......... .C........ .......... .......... .......... .........
T18-1CD7 .......... .......... ..T....... .T........ .......... .......... .......... .......... .......... .......... .......... .......... .......... .......... .......... .......... .C........ .......... .......... .......... .........
T18-1CD8 .......... .......... ..T....... .T........ .......... .......... .......... .......... .......... .......... .......... .......... .......... .......... .......... .......... .C........ .......... .......... .......... .........
M87CD1   .......... .......... ..T....... .......... .......... .......... .......... .......... .......... .......C.. ........G. .......... ......T... .......... .......... .......... .......... .......... .......... .......... .........
M87CD2   .......... .......... ..T....... .......... .......... .......... .......... .......... .......... .......C.. ........G. .......... ......T... .......... .......... .......... .......... .......... .......... .......... .........
M87CD3   .......... .......... ..T....... .......... .......... .......... .......... .......... .......... .......C.. ........G. .......... ......T... .......... .......... .......... .......... .......... .......... .......... .........
M87CD4   .......... .......... ..T....... .......... .......... .......... .......... .......... .......... .......C.. ........G. .......... ......T... .......... .......... .......... .......... .......... .......... .......... .........
M87CD5   .......... .......... ..T....... .......... .......... .......... .......... .......... .......... .......C.. ........G. .......... ......T... .......... .......... .......... .......... .......... .......... .......... .........
M87CD6   .......... .......... ..T....... .......... .......... .......... .......... .......... .......... .......C.. ........G. .......... ......T... .......... .......... .......... .......... .......... .......... .......... .........
M87CD7   .......... .......... ..T....... .......... .......... .......... .......... .......... .......... .......C.. ........G. .......... ......T... .......... .......... .......... .......... .......... .......... .......... .........
M87CD8   .......... .......... ..T....... .......... .......... .......... .......... .......... .........T .......C.. ........G. .......... ......T... .......... .......... .......... .......... ........T. .......... .......... .........
M130CD1  .......... .......... ..T....... .......... .......... .......... .......... .......... .......... .......C.. ........G. .......... ......T... .......... .......... .......... .......... .......... .......... .......... .........
M130CD2  .......... .......... ..T....... .......... .......... .......... .......... .......... .......... .......C.. ........G. .......... ......T... .......... .......... .......... .......... .......... .......... .......... .........
M130CD3  .......... .......... ..T....... .......... .......... .......... .......... .......... .......... .......C.. ........G. .......... ......T... .......... .......... .......... .......... .......... .......... .......... .........
M130CD4  .......... .......... ..T....... .......... .......... .......... .......... .......... .......... .......C.. ........G. .......... ......T... .......... .......... .......... .......... .......... .......... .......... .........
M130CD5  .......... .......... ..T....... .......... .......... .......... .......... .......... .......... .......C.. ........G. .......... ......T... .......... .......... .......... .......... .......... .......... .......... .........
M130CD6  .......... .......... ..T....... .......... .......... .......... .......... .......... .......... .......C.. ........G. .......... ......T... .......... .......... .......... .......... .......... .......... .......... .........
M130CD7  .......... .......... ..T....... .......... .......... .......... .......... .......... .......... .......C.. ........G. .......... ......T... .......... .......... .......... .......... .......... .......... .......... .........
M130CD8  .......... .......... ..T....... .......... .......... .......... .......... .......... .......... .......C.. ...C....G. .......... ......T... .......... .......... .......... .......... .......... .......... .......... .........
```
