# Supplementary figures and images for: Analysis of ancient human mitochondrial DNA from the Xiaohe cemetery: insights into prehistoric population movements in the Tarim Basin, China
Source: BMC Genet. 2015 Jul 8;16:78. doi: 10.1186/s12863-015-0237-5 (PMC4495690; doi:10.1186/s12863-015-0237-5)

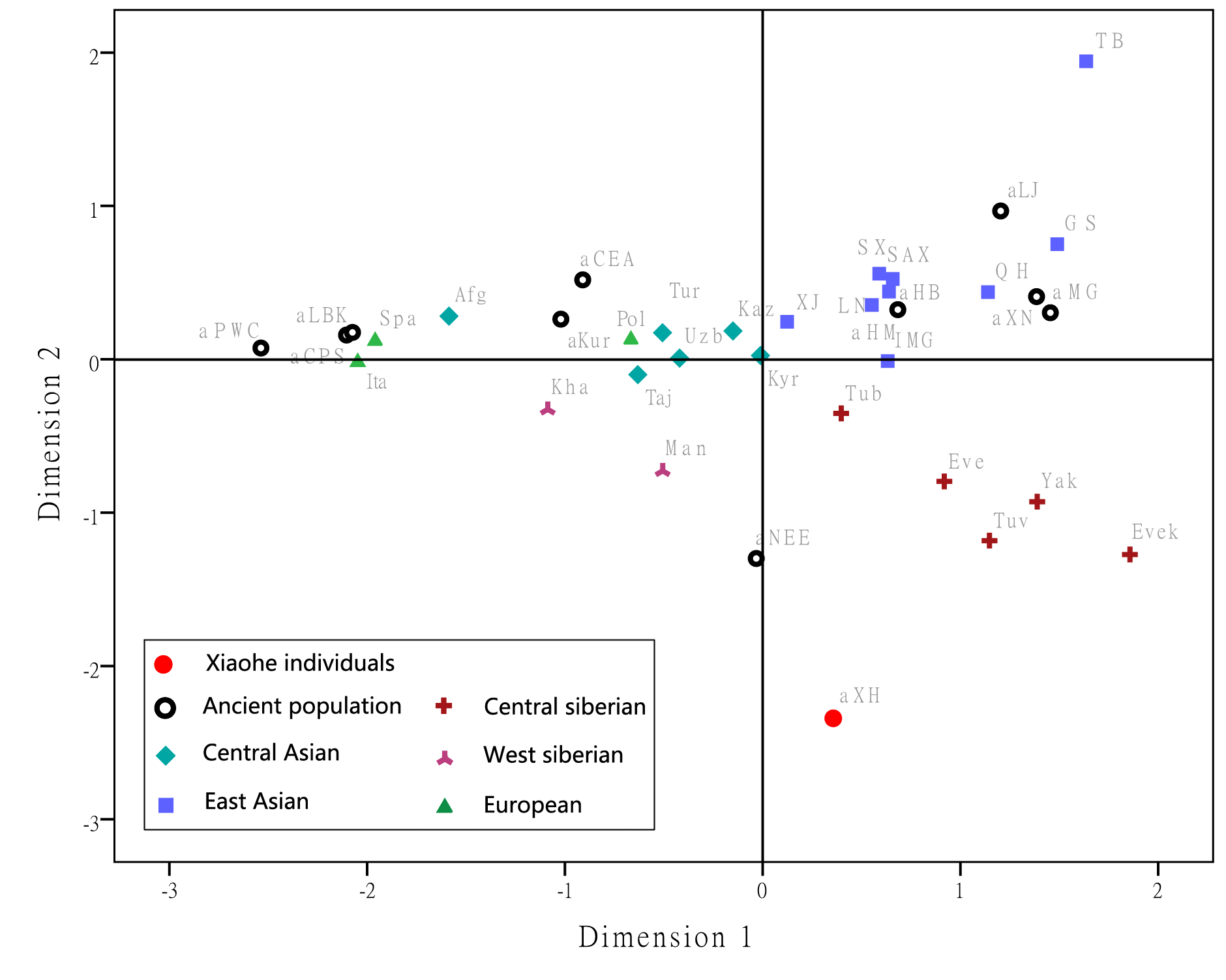

Supplement: Additional file 7: Figure S2. — Multidimensional scaling plot of genetic distances calculated for mtDNA sequences (16050–16391). Population abbreviations are consistent with Fig. 4. [file 12863_2015_237_MOESM7_ESM.tif]
